# New insights into the effects of type and timing of childhood maltreatment on brain morphometry

**DOI:** 10.1038/s41598-024-62051-w

**Published:** 2024-05-18

**Authors:** Yasmin Grauduszus, Maurizio Sicorello, Traute Demirakca, Claudius von Schröder, Christian Schmahl, Gabriele Ende

**Affiliations:** 1 Department of Neuroimaging, Central Institute of Mental Health, Medical Faculty Mannheim, Heidelberg University, Mannheim, Germany; 2 Department of Psychosomatic Medicine and Psychotherapy, Central Institute of Mental Health, Medical Faculty Mannheim, Heidelberg University, Mannheim, Germany

**Keywords:** Post-traumatic stress disorder, Stress and resilience

## Abstract

Childhood maltreatment (CM) is known to influence brain development. To obtain a better understanding of related brain alterations, recent research has focused on the influence of the type and timing of CM. We aimed to investigate the association between type and timing of CM and local brain volume. Anatomical magnetic resonance images were collected from 93 participants (79 female/14 male) with a history of CM. CM history was assessed with the German Interview Version of the “Maltreatment and Abuse Chronology of Exposure” scale, “KERF-40 + ”. Random forest regressions were performed to assess the impact of CM characteristics on the volume of amygdala, hippocampus and anterior cingulate cortex (ACC). The volume of the left ACC was predicted by neglect at age 3 and 4 and abuse at age 16 in a model including both type and timing of CM. For the right ACC, overall CM severity and duration had the greatest impact on volumetric alterations. Our data point to an influence of CM timing on left ACC volume, which was most pronounced in early childhood and in adolescence. We were not able to replicate previously reported effects of maltreatment type and timing on amygdala and hippocampal volume.

## Introduction

Childhood maltreatment (CM) is a pervasive problem. According to world-wide WHO estimates, 3 out of 4 children aged 2 to 4 suffer from regular physical or mental abuse^1^. In western countries such as Germany, the numbers are lower. However, in a recent representative survey^2^, 31% of adults still report one type of child maltreatment.

CM can have lifelong consequences not limited to an increased risk of developing a mental illness^3^ and a higher lifetime prevalence of somatic diseases, including cardiovascular diseases, diabetes, and obesity^4^. Additionally, it has been shown that CM influence both symptom severity and therapy outcome^5,6^.

To be able to provide sustainable support and individualized therapies for victims of CM, it is important to understand the mechanisms underlying neural alterations that result from CM. CM is believed to be responsible for the long-term effects of metabolic adaptation processes and synaptic circuitry in the brain^7^. A high density of glucocorticoid receptors in vulnerable brain regions are considered to be a primary cause of these alterations^8^. Functional changes in the hypothalamic–pituitary–adrenal axis and neurotoxic effects of high cortisol levels are assumed to cause morphological changes^9–11^.

Amygdala, hippocampus, and anterior cingulate cortex (ACC) have frequently been investigated in neurobiological CM research^12^. Although there is consensus about the vulnerability of these regions to stress^13^, reported alterations are ambiguous^12^. Despite earlier studies in animals reporting volumetric increases of the amygdala under the influence of stress^14,15^, a comprehensive meta-analysis showed the opposite effect of decreased amygdala volume after CM in human^16^. For the hippocampus, many studies in animals and humans have demonstrated a smaller volume in those affected by CM. However, here conflicting findings exist as well^16–18^. CM-dependent volumetric alterations of the ACC appear to be more consistent, where studies have mainly shown lower volumes in maltreated humans and also in animals affected by chronic stress^19–22^.

CM is a collective term encompassing a variety of experiences, which may contribute to the diverging results. It can be differentiated regarding several factors, e.g. CM timing, duration, and cumulative effects of different types of CM. Current research is increasingly taking these aspects into account^23,24^. The effects of type on brain morphometry have been repeatedly studied using either type-specific inclusion criteria^20,25^ or subscales of the Childhood Trauma Questionnaire (CTQ)^26,27^. CM can generally be divided into two dimensions: deprivation and threat^28^. “Deprivation” is classified as the lack of necessary influences for cognitive and social development resulting in poorer functional adaptation to complex environments such as one’s social milieu. “Threat” describes actions that are a danger to physical integrity and lead to maladaptive fear responses and deficits in emotion processing^28^. Even though maltreatment often has features of both dimensions, they seem to have differential impacts on brain development resulting in differing morphological alterations^29^. For example, threat appears to result in smaller amygdala volume, while studies focusing on deprivation-like experiences found greater amygdala volume in affected samples^12^.

Besides the type of CM, the developmental period during which CM occurred has also been shown to affect brain morphology and should therefore be given greater attention^24^. It has been suggested that hippocampal stress sensitivity is limited to the postnatal period, while the frontal cortex and amygdala remain sensitive until adolescence or even adulthood^30^. Another study reported reduced hippocampal volume only after repeated sexual abuse in early childhood, whereas changes in the frontal cortex were also observed if sexual abuse occurred in adolescence^31^.

The theory of sensitive periods assumes that regions in the brain go through different developmental phases that are differently susceptible to disturbances such as abusive experiences^32,33^ and has already been investigated in studies on volumetric and functional brain changes as well as clinical outcomes such as dissociative symptoms^34–38^.

**Figure 1:**
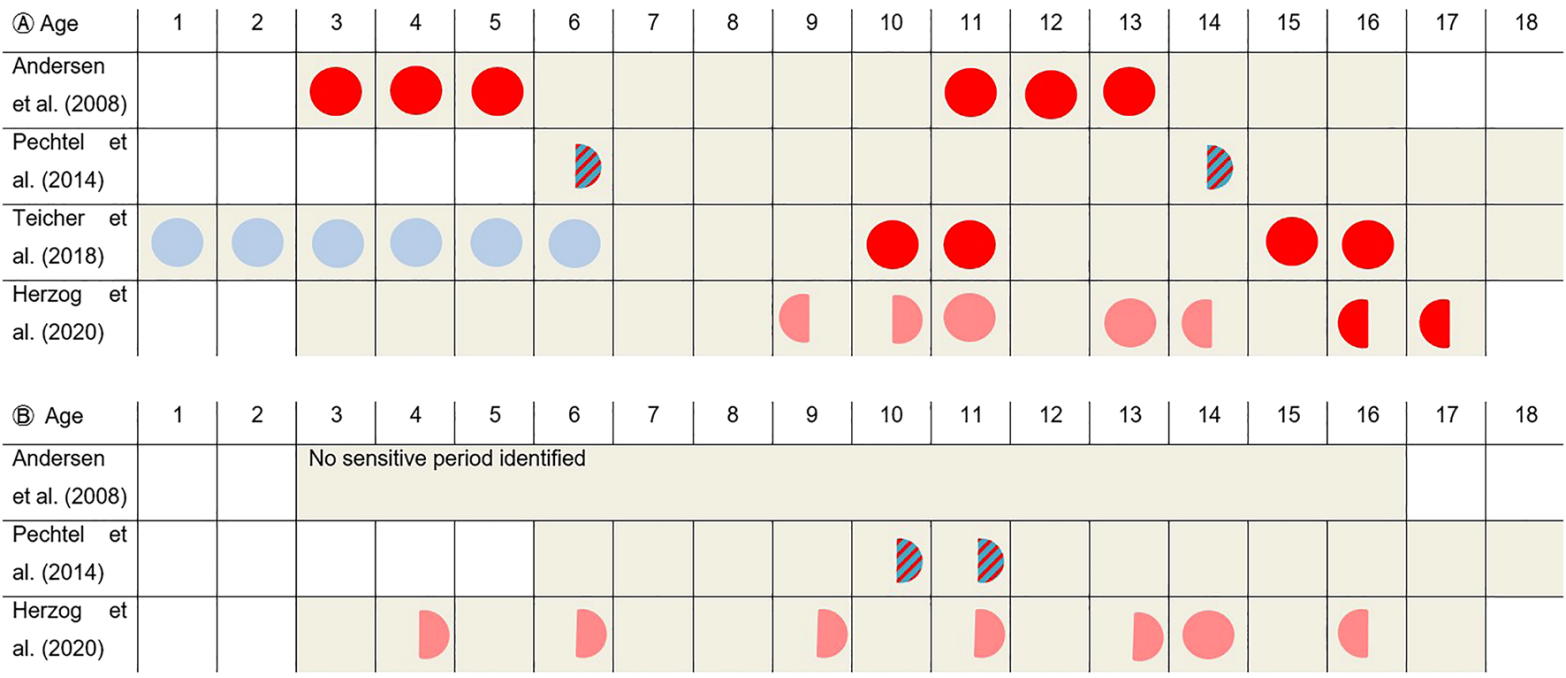
Visualization of key findings from other studies investigating precise timing effects of CM sensitive periods on (**A**) hippocampal volume and (**B**) amygdala. full circle = bilateral, half circle = lateralisation (left or right hemisphere), Type: Abuse = Bold color, Type: Neglect = light color, sex: female = red, sex: male = blue, sex: mixed sample = stripes.

Although several studies have begun to focus on the exact timing of sensitive periods, including the type of CM and sex of the participants, a closer look at the individual results still reveals a complex and inconclusive picture (see Fig. 1a, b). In those studies, vulnerable periods of the hippocampus were mostly pronounced in early childhood, as well as early and late adolescence. Yet, significant differences were observed between men and women when the influence of CM types were accounted for. While one study highlighted that the male hippocampi were only vulnerable to neglect in early childhood and female hippocampi to abuse in adolescence, another one found more prominent vulnerability to neglect in adolescence in their female sample^34,40^.

Concerning the vulnerability of the amygdala (Fig. 1b), it has been demonstrated that the right amygdala is more prone to CM at ages 10 and 11^41^, while another study found that type-specific vulnerability of the bilateral amygdala are more widespread during childhood and adolescence^34^. Regarding the ACC, only one study has investigated sensitive periods^34^: CM at age 10 was an important predictor of left ACC volume and CM at age 3 a predictor of right ACC volume. In the same sample, type-specific abuse severity at age 7 (only left) and neglect severity at age 3–4 (bilateral) figured prominently in ACC volume alterations^34^.

With the present study, our intention was to shed further light on the influence of type and timing of CM on gray matter volume alterations in the hippocampus, the amygdala, and the ACC. These three regions were chosen because they were the most important ones from previous studies with a similar study design. We used a machine learning algorithm with a random forest regression model to identify the variables (i.e. type and timing of CM) with the greatest impact on gray matter volume. To obtain a comprehensive statement about the individual influence of the type and timing of maltreatment, we examined several random forest models with different combinations of type-specific and timing variables. To reproduce the different assumptions and results of the previous studies and to compare our results to these studies, we decided to also review sensitive periods identified from these studies.

## Methods

### Participants

Our study included 93 participants (79 female, 14 male, age 30.9 ± 10,6 years) with a history of self-reported CM within their first 18 years of life (which was the primary inclusion criterion). We included participants who reported at least one event of maltreatment and had a score from at least one point in the “Childhood Trauma Screener” (CTS > 0). An additional 9 participants had to be excluded from the final analysis for various reasons. Two participants showed irregularities in the magnetic resonance (MR) images, caused by movement during the measurement. Another participant was excluded because she was unable to comprehend several important questions during the diagnostic interviews. Six more participants were excluded because clinical data collection could not be completed.

Participants were recruited via advertisements in local newspapers, flyers and the internet. The presence of common mental illnesses that often occur after CM, e.g. PTSD or major depression, was recorded during the study process but was not a factor in the recruitment process, as the study focused on disorder-independent brain alterations after CM. Participants were excluded from the study if they were under the age of 18 or above 60 years of age at the time of the study. Further exclusion criteria included general contraindications for magnetic resonance imaging (MRI) such as large tattoos, pregnancy, metal parts in the body, claustrophobia, etc. None of the participants had a BMI < 17.5 or > 35. Participants with a lifetime psychotic or bipolar disorder (Bipolar-I), as well as a moderate to severe addiction or substance abuse disorder within the last year before participation (> 4 symptoms in SCID-5-CV) were excluded. However, participants with low level of substance abuse (2–3 symptoms in SCID-5-CV), who maintained abstinence within the last 2 months before participation, were included. The use of psychotropic medication two weeks prior participation was another exclusion criterion except for selective serotonin reuptake inhibitors (SSRIs), serotonin reuptake enhancers (SREs), serotonin and norepinephrine reuptake inhibitors (SNRIs) and selective norepinephrine reuptake inhibitors (SNARIs). All participants were required to undergo urine toxicology testing prior to MRI measurement.

This study was part of an ongoing research project within the Research Training Group 2350 (Graduiertenkolleg 2350), funded by the German Research Foundation, that aims to investigate the psychosocial and somatic consequences of childhood maltreatment^42^. It was approved by the Ethical Board II of Heidelberg University, Germany and carried out in accordance with the Declaration of Helsinki at the Central Institute of Mental Health in Mannheim, Germany. Participants were given a thorough explanation of the procedures before providing written informed consent. Each participant was compensated for their participation.

### CM history and health status

To obtain a general history of CM, participants completed the childhood trauma questionnaire CTQ^43^. The CTQ is a widely used self-report measure that captures the severity of the five types of CM: emotional, physical, and sexual abuse, as well as emotional and physical neglect.

The Timing of CM was assessed using the german interview version of the “Maltreatment and Abuse Chronology of Exposure” scale (MACE), called “KERF-40 + ”^44,45^. With this instrument the timing-specific severity for 10 subtypes of CM (8 subtypes of abuse and 2 subtypes of neglect) can be determined and the number of CM subtypes and their duration can be calculated. In addition to the existing KERF-40 + scores, we generated neglect and abuse scores (referred to as “main type”-scores), as well as duration and multiplicity scores for both main types. We decided to differentiate between abuse and neglect as the “main types” and different subtypes e.g. sexual or emotional abuse to account for the different qualities of maltreatment.

For information on (current) psychopathology, participants completed the German versions of the dissociative experience scale (DES; Fragebogen zu Dissoziativen Symptomen^46^), Brief symptom inventory, BSI^47^ and PTSD-Checklist for DSM-V, PCL-V^48^. Psychiatric diagnoses were assessed with the Structured Clinical Interview for DSM-5 SCID-5-CV^49^ by trained doctoral students.

### MRI

High-resolution MRI images were acquired on a 3 Tesla Prisma-Fit scanner (Siemens, Erlangen Germany) using a magnetization-prepared-rapid-acquisition-gradient-echo (“MPRAGE”) sequence: T1-weighted, voxel size 1 × 1 × 1 mm, field of view (FOV) 256 mm, TR = 2000 ms, TE = 2.01 ms, flip angle 9°, 192 slices. During the measurement, participants wore earplugs to reduce background noise.

For further preprocessing, we used the toolbox cat12 (http://www.neuro.uni-jena.de/cat12; Structural Brain Mapping Group, Jena University Hospital, Jena, Germany) in spm12 (Statistical Parametric Mapping, Institute of Neurology, London, UK). First, the images were manually checked for artifacts before the scans were segmented into gray matter volume (GMV), white matter volume (WMV) and cerebrospinal fluid (CSF) using the cat12 toolbox and normalized to the MNI template using the DARTEL algorithm. A homogeneity check was performed as an additional quality measure.

The total intracranial volume (TIV = gray matter GM + white matter WM + cerebrospinal fluid CSF) is automatically estimated in the segmentation process of cat12. The volumes of the regions of interest (ROI) were estimated and extracted in cat12 using the “Neuromorphometrics Atlas” (Neuromorphometrics, Inc.).

### Statistics

All clinical data, as well as the estimated ROI-volume from both left and right amygdala, hippocampus, and ACC, were imported into SPSS (IBM SPSS Statistics 27) for further processing.

Due to the high dependence of ROI volume on whole−brain volume, their proportion relative to total intracranial volume (TIV = gray matter + white matter + cerebrospinal fluid) was first calculated and then corrected for using participant age with a regression analysis. The residuals were z-transformed around the center 100 ± 10.

### Random forest regression

Random forest regressions with conditional interference trees were performed (“cforest” in the R package “party”) to determine which variables of CM had the most explanatory power in detecting morphological brain alterations^50,51^.

Specifically, the algorithm was used to find out whether the severity of maltreatment in individual developmental years was associated with brain alterations or weather other maltreatment characteristics like types or duration have a higher association. This is a common methodology for analyzing sensitive periods^40,41^.

This machine-learning algorithm is used to predict a dependent variable by a set of given independent variables (“predictor variables”) and has been repeatedly used for similar approaches^34,40,41^. The algorithm creates a set of unique decision trees, which are combined for an overall result and provide information on the predictive accuracy of the whole model as well as the variable importance of each predictor variable. Detailed information can be found in the Supplementary Materials.

We created 6 models with a different set of predictor variables, starting with the basic scores (severity, duration, number of subtypes). Subsequent, more detailed models additionally included timing-specific severity scores. All predictor variables used in the random forest analysis resulted from the KERF-40 + . For the examination of timing-specific CM, our analysis included ages 3 and older, since autobiographical memory is known to be unreliable before age 3^52^. To test whether the sex of the participants had an impact on the outcome of the random forest model, sex was used as an additional covariate in each model.

The models were:

- model 1: Basic model-CM.
- model 2: Timing of CM.
- model 3: Basic model of main types (neglect & abuse).
- model 4: Timing of main types.
- model 5: Subtype model.
- model 6: Timing of subtypes.

The analysis was performed in three steps:

- (i) In R, the random forest models were first tested individually for each ROIs mean accuracy (calculation of the cross-validated R^2^ based on the out-of-bag samples)^53^.
- (ii) Model/ROI combinations without predictive value (R^2^ < 0) were excluded from further steps. Variable importance values were then calculated for model/ROI combinations with a positive accuracy using the random forest analyses.
- (iii) Variables identified as important in the random forest analyses were entered in a correlation analysis along with our chosen dependent variable, to determine the direction of the relationship between them.

### Verification of the sensitive periods from other studies

The present study attempted to replicate the results of other studies with our sample. In addition to our own random forest analysis, sensitive periods revealed in other studies were reviewed. For this purpose, we evaluated the timing parameter identified as sensitive periods for the amygdala, hippocampus and anterior cingulate cortex of these three prior studies: Pechtel et al.^41^, Teicher et al.^40^ and Herzog et al.^34^. These three studies were chosen as they had a similar study design. i.e.: (a) used the MACE/KERF for assessing CM, (b) used random forest regression to identify sensitive periods, and (c) focused on morphological brain alterations.

Since each of these studies used a different set of control variables (age, sex, and brain volume), we performed each analysis twice using different combinations of covariates. First, correlation analyses were performed as close as possible to the conditions of the corresponding study (identical covariates, etc. so called analog (to original) version). In a second analysis, we used the same combination of covariates as in our random forest analysis (relative volume & corrected for age, see also Section Random Forest, so called Mannheim version).

#### Pechtel, et al.^41^

For the analog version, the volumes of the right and left amygdala were corrected by calculating the proportion of brain gray matter volume (GMV). Correlations were calculated with the GMV-corrected amygdala volumes and overall CM severity (KERF-40 + sum), as well as CM severity at ages 10 and 11 years. In addition, the right amygdala volume in the quartile with the highest CM severity at age 11 years was compared against the quartile with the lowest CM severity at that age. Correlations between GMV-corrected hippocampal volumes and CM severity at ages 7 and 14 years were also calculated. For the Mannheim version, the TIV- and age-corrected values were then used.

#### Teicher, et al.^40^

For the analog version, we summed the side-specific hippocampal volumes to obtain a value for bilateral hippocampal volume. We used the raw values for correlation analysis*.* In the Mannheim version, the TIV and age-corrected value were then used (see also Section Random Forest). Initially, neglect at age 7 and abuse at ages 15 and 16 were examined for the entire sample. Subsequently, the sample was separated by sex. Abuse at ages 10,11,15,16 and multiplicity were examined for the female subsample and neglect at ages 1 to 7, as well as multiplicity were examined for the male subsample.

#### Herzog, et al.^34^

Following the original study, only the female subsample was used for the replication. In addition, we created additional (timing-specific) abuse scores by summing the two sexual abuse scores together with the physical abuse score and calculating the mean value, as they only used these scores for their abuse score. Correlation analyses were thus performed with all predictor variables that were also tested by Herzog et al. A detailed description of all predictor variables can also be found in title S8 in the Supplements from Herzog, et al.^34^.

## Results

### CM history

**Figure 2:**
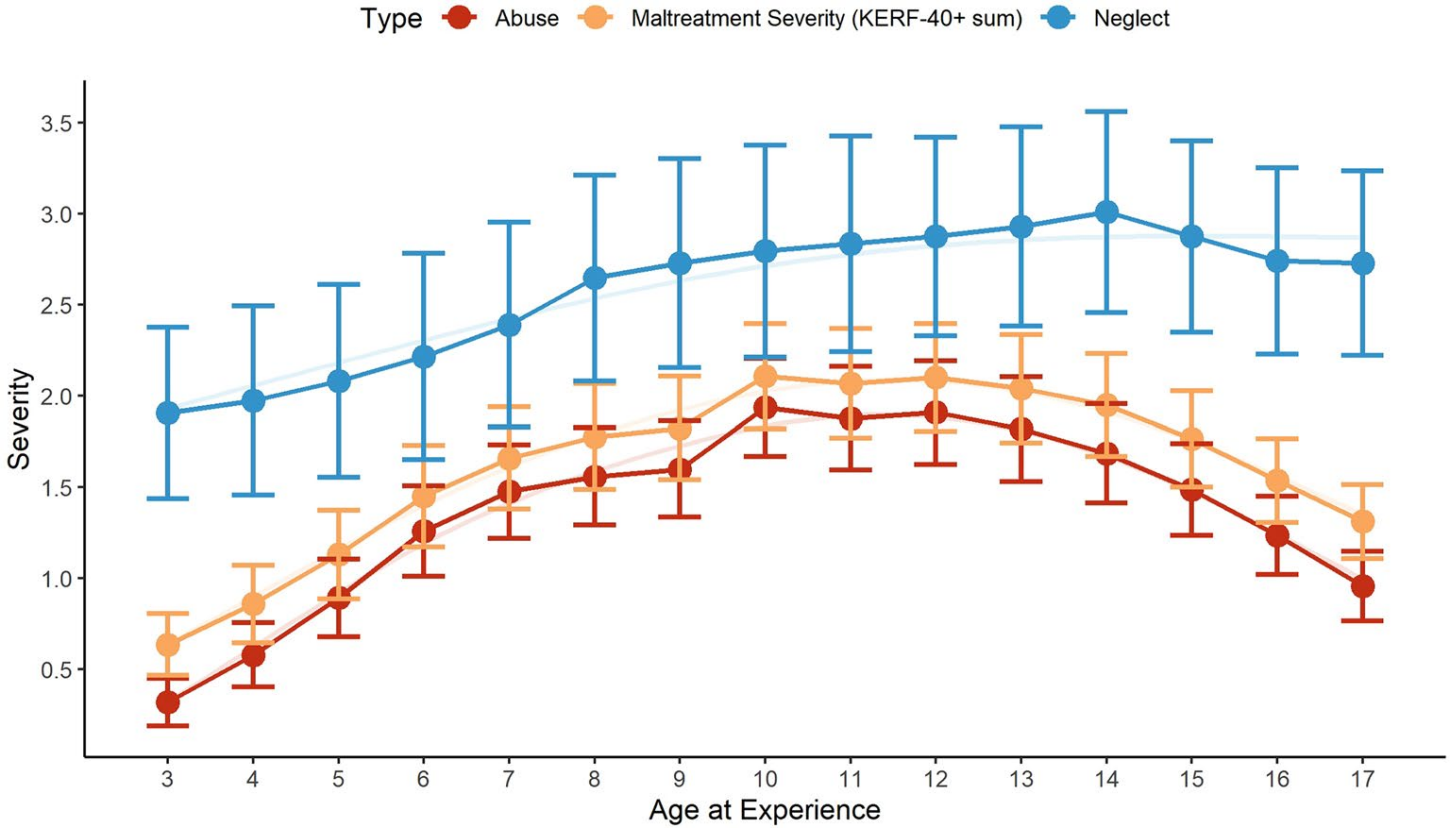
Distribution of CM severity across ages 3–17; for clarity of presentation, overall CM severity (KERF-40 + sum score, yellow) was adjusted to a scale 0–10 (severity/10 = value on y-axis).

Participants’ exposure to abuse, neglect, and CM in general across time can be seen in Fig. 2. Abuse peaked between 10 and 12 years of age, neglect later at 14 years of age.

Participants in our sample suffered primarily from parental emotional abuse (n = 60), peer abuse (n = 46), physical abuse (by parents, n = 40), and emotional neglect (n = 65). Even though the mean severity score of exposure to sexual abuse was lower in comparison to these subtypes, more than half of the participants had experienced some kind of sexual abuse outside of the household (n = 53, see Table 1).

**Table 1:** CM characteristics KERF-40+ , all subscale scores (including total count of participants over cutoff) and the three global characteristic values (with splitting into main types).

n = 93	Mean	SD	Over cutoff (n)
**KERF-40 + subscales/subtypes**			
Parental emotional abuse (PEA)	5.5	2.7	60
Physical & emotional abuse by siblings (PEAS)	1.8	2.8	16
Physical & emotional abuse by peers (PEER)	4.9	3.4	46
Witnessed violence towards siblings (WITS)	3	3.4	29
Parental physical abuse (PPA)	5.5	3.6	40
Emotional neglect (EN)	6.0	3.9	65
Physical neglect (PN)	2.9	3.1	31
Witnessed violence towards parents (WITP)	1.7	2.5	17
Sexual abuse by a member of the household (SEXA_H)	1.0	2.0	24
Sexual abuse by others not living in the same household (SEXA_O)	1.8	1.9	53
**KERF-40 + sum (overall severity)**	34.1	15.5	
Abuse	3.1	1.4	
Neglect	4.5	3.2	
**KERF-40 + duration (in years)**	12.2	5.3	
Abuse	9.1	4.9	
Neglect	7.9	7.3	
**KERF-40 + multiplicity (number of subtypes)**	4.1	2.0	
Abuse	3.1	1.6	
Neglect	1	0.8	

Additional information on CM history and mental health status can be found in the supplementary material.

### Random forest

#### Accuracy

This step was the preceding analysis to test the overall predictive accuracy of the models and their significance, which is a prerequisite for further random forest analyses. For the bilateral hippocampus and amygdala, the accuracy check revealed negative values for each model. Testing the accuracy of the models for prediction of the right and left ACC volumes revealed only one predictive model for each ROI, with model 1 predicting the right ACC volume and model 4 predicting left ACC volume.

For model 1, a model with basic CM characteristic scores, predicting right ACC volume, we found a positive, albeit small, accuracy R^2^ = 0.011 (p = 0.043). For model 4, a model with both type-specific timing values and basic neglect and abuse scores that predicted the left ACC volume, we found a positive, but also small accuracy R^2^ = 0.011 (p = 0.042).

#### Calculation of importance of predictor variables

For further clarification of the impact of the type and timing of CM, the influence of each variable in the predicting models was examined.

##### Right ACC

**Figure 3:**
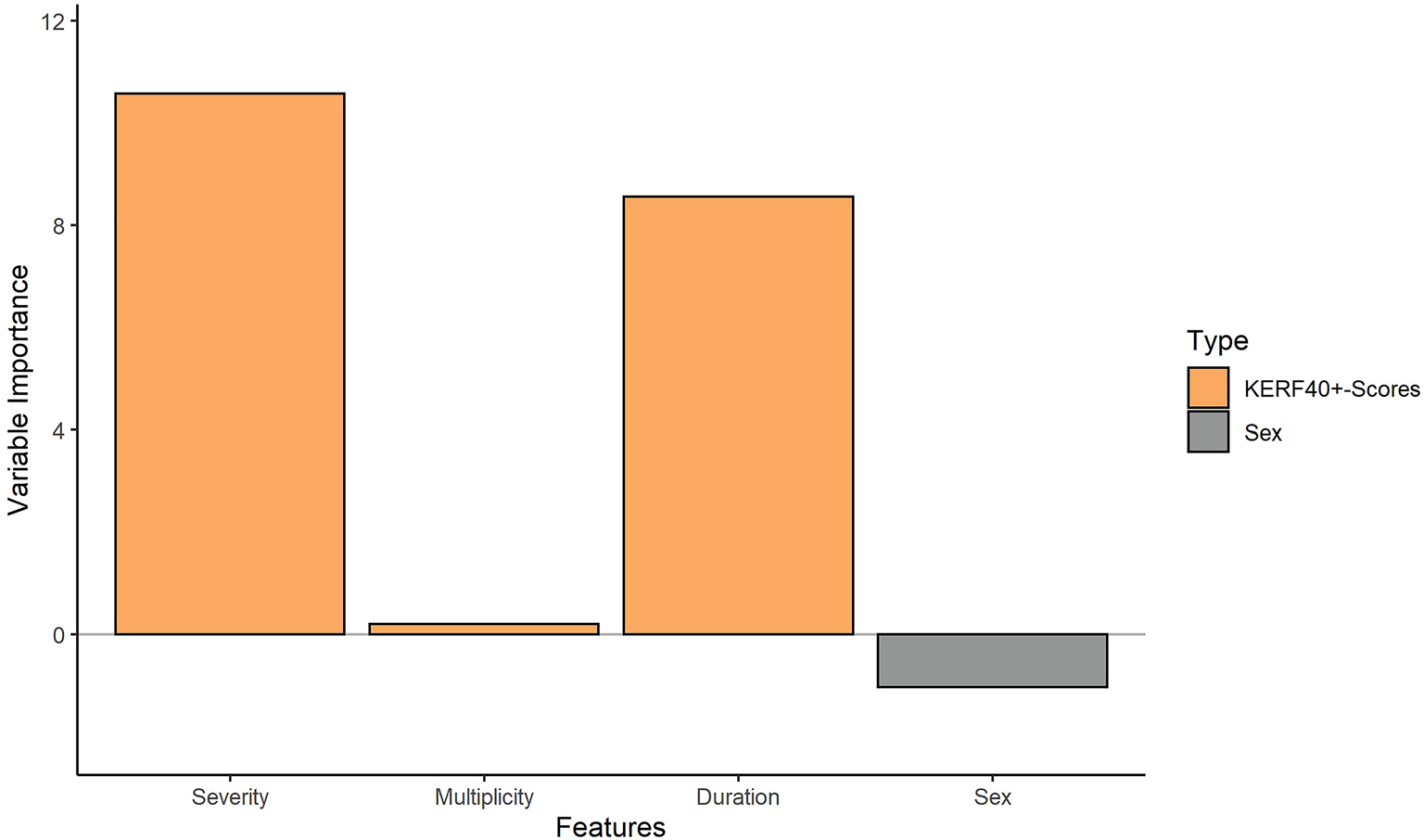
Results of the random forest regression of model 1 for the right ACC, showing the variable importance for each variable of the model. Severity = KERF-40 + sum, Multiplicity = KERF-40 + multiplicity, Duration = KERF-40 + duration.

The calculation of variable importance (VI) parameters revealed that overall CM severity (VI = 10.58, p_uncorr_ = 0.012, p_FDR-corr_ = 0.048) and CM duration (VI = 8.56, p_uncorr_ = 0.041, p_FDR-corr_ = 0.082) were important predictors of the right ACC volume (see Fig. 3). Sex showed no predictive importance (VI = −1.04, p_uncorr_ = 0.78). In a correlation analysis, both overall CM severity (KERF-40 + sum) and duration showed no significant association with right ACC volume.

##### Left ACC

**Figure 4:**
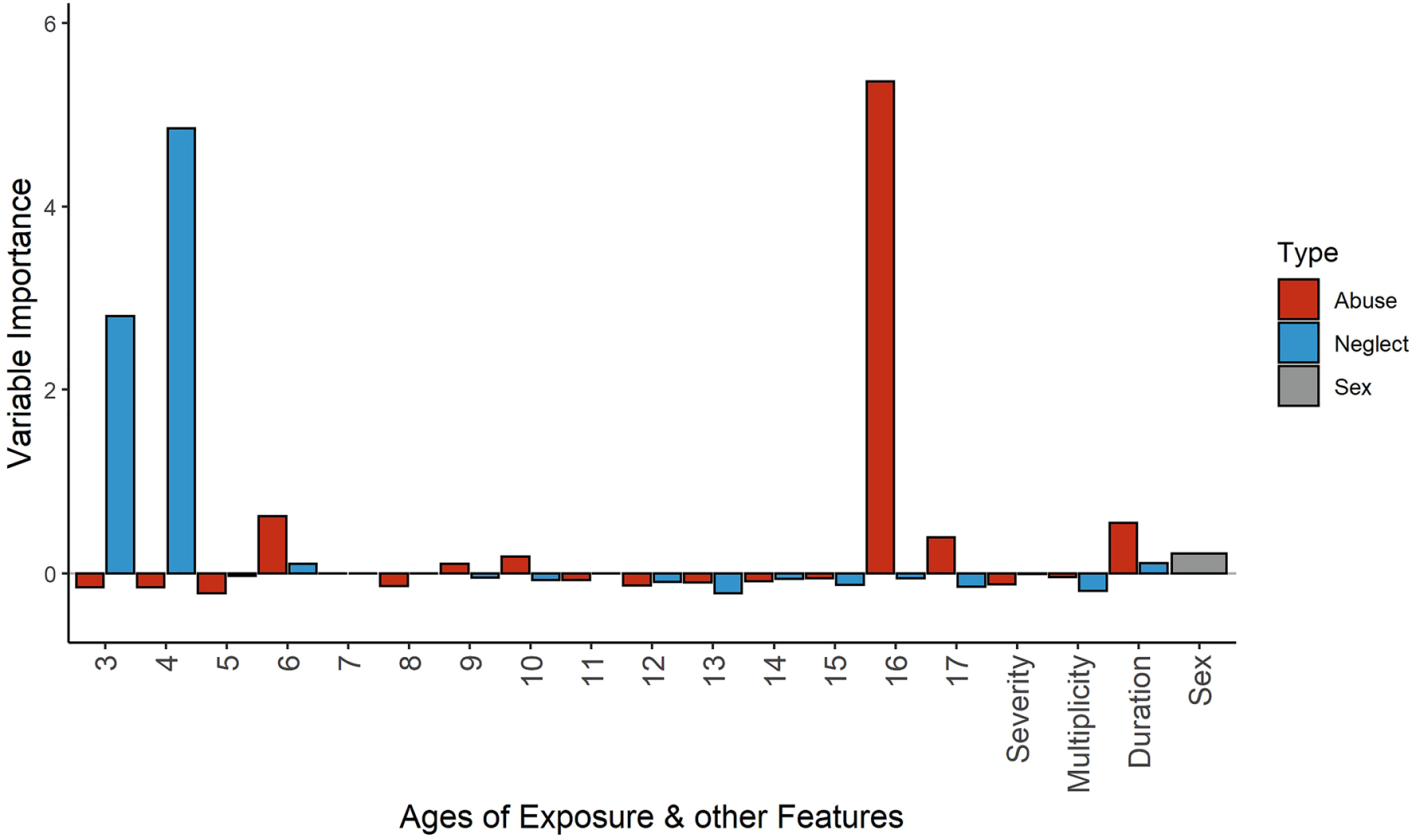
Results of the random forest regression of model 4 for the left ACC, showing the variable importance for each time-specific severity scores for neglect and abuse as well as the other features of the model.

The calculation of variable importance revealed that abuse severity at age 16 had the highest importance for left ACC volume: VI = 5.36 (p_uncorr_ = 0.005, p_FDR-corr_ = 0.093, see Fig. 4). Other important predictor variables were neglect severity at ages 3 (VI = 2.8, p_uncorr_ = 0.016, p_FDR-corr_ = 0.197) and 4 (VI = 4.85, p_uncorr_ = 0.004, p_FDR-corr_ = 0.093). Sex revealed no predictive importance (VI = 0.21, p_uncorr_ = 0.12). In a correlation analysis, neglect at ages 3 and 4 years showed a significant positive association with the volume of the left ACC. For abuse severity at age 16, a significant association with the volume of the left ACC could not be found in a correlation analysis.

### Verification of the sensitive periods from other studies

#### Pechtel et al.^41^

For the analog version, GMV-corrected right hippocampal volume showed a positive correlation with overall CM severity (r = 0.243 p = 0.019) as well as CM severity at age 7 and 14 (7: r = 0.28 p = 0.021, 14: r = 0.288 p = 0.005). The Mannheim-version with the volumes corrected for total intracranial volume and age did not result in significant correlations (see Table 2).

**Table 2:** Correlation analyses of timing-specific severity scores according to Pechtel et al. (2014) with ROI volume.

		Mannheim-version		Analog-version	
		Pearson r	Sign. p	Pearson r	Sign. p
Amygdala right	KERF-40 + sum	0.036	0.733	0.201	0.053
	KERF-40 + sum age 10	0.007	0.951	0.168	0.106
	KERF-40 + sum age 11	0.033	0.753	0.192	0.065
Hippocampus right	KERF-40 + sum	0.056	0.592	0.243*	**0.019**
	KERF-40 + sum age 7	0.062	0.555	0.238*	**0.021**
	KERF-40 + sum age 14	0.165	0.113	0.288*	**0.005**

*p < 0.05.

Significant values are in bold.

#### Teicher et al.^40^

No significant correlations were found in the total sample, nor in male or female subsamples using the original methodology (analog-version). After correcting the dependent variable for whole brain volume (TIV) and age, again no significant correlation could be identified (Mannheim-version, see Table 3).

**Table 3:** Correlation analyses of timing-specific severity scores according to Teicher et al. (2018) with ROI volume.

		Mannheim-version		Analog-version	
		Pearson r	Sign. p	Pearson r	Sign. p
Total sample (n = 93)	Neglect age 7	0.162	0.121	0.106	0.31
	Abuse age 15	−0.017	0.871	−0.041	0.694
	Abuse age 16	0.002	0.986	0.011	0.918
Female (n = 79)	Abuse age 10	0.028	0.805	−0.21	0.063
	Abuse age 11	0.027	0.816	−0.19	0.093
	Abuse age 15	−0.061	0.595	−0.049	0.669
	Abuse age 16	−0.03	0.792	−0.016	0.889
	KERF-40 + multiplicity	0.056	0.850	0.276	0.340
Male (n = 14)	Neglect age 1	−0.103	0.727	0.333	0.245
	Neglect age 2	−0.009	0.975	0.306	0.287
	Neglect age 3	−0.009	0.975	0.306	0.287
	Neglect age 4	−0.05	0.865	0.171	0.559
	Neglect age 5	−0.005	0.988	0.28	0.332
	Neglect age 6	−0.003	0.992	0.439	0.116
	Neglect age 7	0.047	0.872	0.408	0.148
	KERF-40 + multiplicity	0.040	0.726	-0.120	0.294

*p < 0.05.

#### Herzog et al.^34^

Since Herzog et al. used the same correction for cranial volume and age, mainly results of the Mannheim-version are shown. Only sensitive periods of abuse have been tested in two versions. The left ACC showed a correlation with timing-specific severity of neglect at ages 3 and 4 (ages 3: r = 0.233, p = 0.039, and ages 4: r = 0.282, p = 0.012) in the female cohort (see Table 4).

**Table 4:** Correlation analyses of timing-specific severity scores according to Herzog et al. (2020) with ROI volume.

		Mannheim-version		Analog-Version	
		Pearson r	Sign. p	Pearson r	Sign. p
Amygdala left	KERF-40 + sum age 13	0.167	0.142		
	Neglect age 14	0.096	0.399		
	Neglect age 16	0.117	0.306		
Amygdala right	KERF-40 + sum age 10	0.016	0.888		
	KERF-40 + sum age 13	0.065	0.568		
	Neglect age 4	0.194	0.087		
	Neglect age 6	0.088	0.439		
	Neglect age 9	0.111	0.329		
	Neglect age 11	0.098	0.393		
	Neglect age 13	0.172	0.13		
	Neglect age 14	0.138	0.226		
Hippocampus left	KERF-40 + sum age 10	0.09	0.429		
	KERF-40 + sum age 11	0.106	0.351		
	KERF-40 + sum age 13	0.131	0.249		
	Neglect age 9	0.151	0.185		
	Neglect age 11	0.097	0.397		
	Neglect age 13	0.166	0.144		
	Neglect age 14	0.168	0.139		
	Abuse age 16	−0.043	0.706	−0.157	0.167
	Abuse age 17	0.006	0.955	−0.101	0.377
Hippocampus right	KERF-40 + sum age 10	0.018	0.872		
	KERF-40 + sum age 11	0.009	0.938		
	KERF-40 + sum age 13	0.101	0.377		
	Neglect age 10	0.088	0.441		
	Neglect age 11	0.085	0.456		
	Neglect age 13	0.221	0.051		
ACC left	KERF-40 + sum age 10	0.198	0.08		
	Neglect age 3	0.233*	**0.039**		
	Neglect age 4	0.282*	**0.012**		
	Abuse age 7	0.01	0.933	0.155	0.173
ACC right	KERF-40 + sum age 3	0.071	0.533		
	Neglect age 3	0.092	0.418		
	Neglect age 4	0.144	0.206		

Right column: with specific abuse score only with physical and sexual abuse (see method section).

*p < 0.05.

Significant values are in bold.

## Discussion

The present study investigated the impact of CM on amygdala, hippocampus, and ACC morphology using random forest regression models. A particular focus was on whether the inclusion of type- and timing-specific CM severity adds value to explaining volumetric changes in these regions or, whether looking solely at purely cumulative scores such as overall severity, number of subtypes, or duration provides better explanations. In addition, we tested stepwise whether increasing differentiation between CM main types and subtypes can provide better prediction.

Our findings indicate maltreatment-dependent ACC volume alterations. The results suggest that the main type-specific timing of CM has an impact on left ACC volume, with neglect in early childhood and abuse in adolescence having the greatest impact. We discovered that there was a positive association between the timing-specific effects of neglect and left ACC volume. These results confirm the sensitive period of the ACC to neglect previously identified by Herzog, et al.^34^

The pattern of a very early and a late (adolescent) sensitive period has also been demonstrated in studies on amygdala activity^37^. In addition, the pattern of vulnerability to neglect in early childhood and to abuse in adolescence has also been demonstrated in the hippocampus^40^. A possible explanation is that due to the increasing autonomy of the individual in adolescence, neglect has a significantly greater influence on the young child. An enlarged ACC was found in a study of children aged around 13 years with neglect as the only form of maltreatment experienced compared to children without maltreatment experiences^54^. In line with this, a recent meta-analysis has shown that the direction of brain volume alterations, e.g. in ACC and hippocampus, changes from an increase in volume in childhood to a decrease after the age of 12 in subjects with interpersonal adversities^55^. Assuming this change in direction of influence exists, it may be difficult to retrospectively detect the influence of maltreatment if subjects experienced both childhood and adolescence maltreatment. This may be one reason for the small variance that can be explained by our models, and which must be considered when interpreting the results.

Main and Sub-type or timing did not predict right ACC volume, although general CM characteristics such as overall severity and duration did. In Herzog, et al.^34^, overall CM severity also showed an important effect on ACC volume, albeit in the left hemisphere. To our knowledge, the effect of duration on ACC volume has not been investigated in studies to date.

In contrast, none of the random forest models were predictive of volumetric alterations specific to the amygdala and hippocampus in our sample. This contrasts with previous studies, where CM-dependent alterations in amygdala and hippocampal volume, including timing-dependent ones, have been repeatedly reported^24^. Reasons for this may be manifold. It should be considered that the reported sensitive periods of previous studies, as well as their methodology, also differed^30,31,39^. Even in those studies using random forest regression, sample composition and consideration of covariate differed^34,40,41^. Therefore, we considered it even more important to replicate the results of the previous studies in a relatively large sample.

For this reason, we not only performed random forest analyses, but also tested the sensitive periods identified in other studies using covariates analog to these studies in our sample. We found significant correlations in the analog version between right hippocampal volume and the overall CM severity and CM-severity at ages 7 and 14 (KERF-40 + sum) taken from the Pechtel et al.^41^ study. Beyond this, only a few statistical trends emerged. The Mannheim version revealed different results, due to the inclusion of TIV and age as essential covariates.

With our study, we tried to find the most important predictors for volumetric alterations by combining other studies’ methodology and testing the accuracy of different explanation approaches. In contrast to other studies, we decided to first examine and compare models of overall and specific predictor variables for their explanatory power, to obtain an estimation of whether increased differentiation (e.g. of CM types) is meaningful before checking for individual variable importance. This also ensured that no unimportant variable was considered important based on poor quality of the overall model. We evaluated both sensitive periods for CM as a whole and depending on the main and subtype of maltreatment.

For the generation of the scores, we decided, following Teicher, et al.^40^, to include all subtypes of abuse for the abuse score. Herzog, et al.^34^ generated their abuse scores only from the sexual and physical abuse scores, which were also a primary inclusion criterion in their study. Thus, the sensitive periods they found may be very specific to these two subtypes of abuse. According to our results, the question remains how informative the differentiation into main types and subtypes is when describing volumetric changes of the hippocampus and amygdala, because none of the models demonstrated explanatory power. The question of whether different sensitive periods exist for the sexes also remains open. In the literature, different findings have been reported. While one study was able to demonstrate a larger effect of neglect in women^34^, another study demonstrated only sensitive periods for abuse in women and only for neglect in men^40^.

There are a number of limitations that must be taken into account when considering the results of this work.

CM history was reported retrospectively. Details of events, such as exact timing of CM, are often difficult to remember and possibly distorted. The recording of other important events, e.g., school events, moves, etc., at the beginning of the interview served as a measure of control to anchor the participants’ memories in their personal history. However, it should be noted that an investigation of the reliability of the KERF-40 + revealed high reliability values^44^.

We tried to recruit equal numbers of male and female participants in our sample to be able to make general statements and identify possible differences. However, mainly women volunteered to participate in the study. Although the prevalence of stressful events is thought to be similar in both men and women, men are less likely to report these experiences and are often underrepresented in studies of mental illness. In favor of a higher sample size, we decided to tolerate this asymmetry in the distribution. However, the interpretation of the significance of brain alterations in men is limited.

Lastly, the statistical power of the design is essential for understanding the implications of the null findings we report. Sample size requirements of the random forest approach are, in principle, similar to those of traditional linear models, while being arguably slightly better due to its (on average) better model fit^56,57^ as well as its relative insensitivity to multicollinearity and the number of predictors^58^. In accordance with this notion, we recently observed that the power of the cross-validated variance explained was similar to that observed using gpower for linear regression R^2^ when predicting amygdala function from KERF data^36,37^. At the same time, its advantages make random forests well-suited for the identification of sensitive periods, as has been previously shown^59^. Given that sample size requirements for R^2^ are often similar for random forest and linear regression, this leaves the question whether our design has sufficient statistical power to be meaningful. In previous similar random forest studies, except in two studies^36,37^, it does not seem to be commonplace to report R^2^, which we consider important for contextualizing the results and see as a strength of our work to be transparent in this regard. As we already conducted a previous study on 68 participants^34^, which found statistically significant effects, our main goal was to increase sample size beyond this number as much as possible within the project timeline. This led to our sample of 93 participants, which we believe is an appropriate increase for a conceptual replication. Moreover, beyond the random forest approach, we included simple bivariate linear models for previously reported sensitive life years to facilitate the accumulation of evidence between studies.

Taken together, this study examined the influence of type and timing of CM in a sex-mixed sample to better understand volumetric alterations in sensitive brain regions as a result of these experiences. Therefore, we created a comprehensive methodology to test different approaches with and without type specific and timing-specific CM severity. We were able to verify the influence of neglect in early childhood and abuse in late adolescence on ACC volume but were not able not explain volumetric alterations of the hippocampus and amygdala with our models. We consider it important that further studies with a larger number of participants investigate the influence of type and timing. Currently, there are large differences between studies, both in terms of their results and methodology. There is also an increasing number of studies with a longitudinal study design, which have the possibility to follow development more directly, but are dependent on other aspects such as the difficulty of recording adverse events in minors by third parties^60,61^. Aggregation of the results of different studies may help to reveal findings and get a better picture of the reality. We propose a stepwise approach with different variable combinations, as we have implemented here, to get more information about the impact of different main- and subtypes on volume alterations.

### Supplementary Information

Supplementary Information.

## Data Availability

The datasets generated during and analyzed during the current study are available in the OSF repository, https://osf.io/u98va/?view_only=73eaa482e1274522b25811a558f3ca3a.

## Code availability

The Analysis code is available in the OSF repository, https://osf.io/u98va/?view_only=73eaa482e1274522b25811a558f3ca3a.
