# Supplementary material for: New insights into the effects of type and timing of childhood maltreatment on brain morphometry
Source: Sci Rep. 2024 May 18;14:11394. doi: 10.1038/s41598-024-62051-w (PMC11102438; doi:10.1038/s41598-024-62051-w)
Supplement: Supplementary file 1 — Supplementary Information. [file 41598_2024_62051_MOESM1_ESM.pdf]

1 Supplement

1.1 Title

New insights into the effects of type and timing of childhood maltreatment on brain morphometry

1.2 Authors & Affiliations:

1. Yasmin Grauduszus*[1], yasmin.grauduszus@zi-mannheim.de
2. Maurizio Sicorello[2], maurizio.sicorello@zi-mannheim.de
3. Traute Demirakca[1], traute.demirakca@zi-mannheim.de
4. Claudius von Schröder[2], claudius.schroeder@zi-mannheim.de
5. Christian Schmahl[2], chrstian.schmahl@zi-mannheim.de
6. Gabriele Ende[1], gabi.ende@zi-mannheim.de

[1]Department of Neuroimaging, Central Institute of Mental Health Mannheim, Medical Faculty Mannheim, Heidelberg University, Germany
[2]Department of Psychosomatic Medicine and Psychotherapy, Central Institute of Mental Health, Medical Faculty Mannheim, Heidelberg University, Germany

Christian Schmahl and Gabriele Ende contribute equally.

1.3 Methods

1.3.1 Random Forest

Random-forest regressions were performed in R Studio (version 4.1.3). Per random forest, the algorithm created 500 unique decision trees. Each tree is generated by a subsample of the entire dataset and the predictive performance of the model is estimated from the omitted sample (out-of-bag sample). Aggregating the different trees improves the predictive power of the whole forest [1].

The advantage of this methodology over other linear modeling and procedures is a better differentiation in case of multicollinearity between predictor variables as well as the independence of the number of predictors and distribution assumptions. Non-linear relationships can also be identified in random forest regression. It should be considered that the result of a random forest regression provides no information on the nature of the relationship between the important variables and the dependent variables.

The importance of each variable for the prediction performance is calculated by decreasing the model accuracy after permuting each predictor variable and can be seen in the “variable importance” (VI). The variable importance is calculated for each predictor variable. Variables that are important for the prediction model, lead to a decrease in model accuracy when they are permuted. If the permutation of a variable leads to only small changes in model accuracy, it is classified as "unimportant" [2].

For stability, each random forest regression was repeated 20 times and mean values for accuracy and variable importance’s were calculated.

To assess statistical significance, permutation tests were performed by permuting the dependent variables 1000 times per model. The p-value is the proportion of permutation-based variable importance values that are greater or equal than the non-permuted value [3].

Variables in the models:

model 1	KERF-40+ sum, KERF-40+ duration, KERF-40+ multiplicity, sex
model 2	model 1plus KERF-40+ sum for each age 3-17 years
model 3	overall abuse severity, overall neglect severity, duration of abuse, duration of neglect, number of abuse subtypes, number of neglect subtypes, sex
model 4	model 4 plus abuse severity for each age 3-17 plus neglect severity for each age 3-17
model 5	SUM_PEA, SUM_PPA, SUM_PEAS, SUM_EN, SUM_PN, SUM_WITP, SUM_WITS, SUM_PEER, SUM_SEXA-H, SUM_SEXA-O, sex
model 6	model 5 plus all age-specific severity scores (age 3-17) of all subtypes

1.4 Results

1.4.1 ACE History and Health Status

The distribution of neglect subtypes is quite regular with 27 participants having experienced no neglect, 36 participants having experienced one of the two neglect subtypes and 30 participants both subtypes (emotional and physical). Most participants suffered trauma from three different KERF-40+ abuse subtypes (n=26), 34 participants from four or more subtypes, 33 participants suffered trauma from less than three abuse subtypes.

The duration of how long participants were affected by ACE is best considered separately by type because of the very different distribution patterns. About half of the participants either experienced no neglect (n=28) or experienced neglect throughout their whole childhood and adolescence (n=22). The duration of abuse showed a more regular distribution over each duration.

At the time of participation, about one third of participants suffered from a post-traumatic stress disorder (n=27), an affective disorder (n=27) and/or an anxiety disorder (n=35). 77 participants suffered from at least one disorder of these three disorder groups during their life.

Supplementary Table S1: CM characteristics KERF-40+, all subscale scores (including total count of participants over cutoff) and the three global characteristic values (with splitting into main types) divided by sex

n_{female}=79, n_{male}=14	mean		SD	
	Fem.	Male	Fem.	Male
**KERF-40+ Subscales/Subtypes**				
Parental Emotional Abuse (PEA)	5.5	5.3	2.6	3.1
Physical & Emotional Abuse by Siblings (PEAS)	1.7	2.3	2.7	3.2
Physical & Emotional Abuse by Peers (PEER)	4.9	5.4	3.3	4.1
Witnessed Violence towards Siblings (WITS)	2.7	4.3	3.3	3.7
Parental Physical Abuse (PPA)	5.3	6.8	3.5	4.1
Emotional Neglect (EN)	5.9	6.4	4	3.9
Physical Neglect (PN)	2.7	3.9	3	3.6
Witnessed Violence towards Parents (WITP)	1.7	1.6	2.3	3.3
Sexual Abuse by a Member of the Household (SEXA_H)	1.1	0.5	2.1	1
Sexual Abuse by Others Not Living in the Same Household (SEXA_O)	2.1	0.3	2	0.8
**KERF-40+ sum (overall severity)**	**33.6**	**36.8**	**15.1**	**17.9**
abuse	3.1	3.3	1.4	1.7
neglect	4.1	5.2	3.1	3.4
**KERF-40+ Duration (in years)**	**12.1**	**12.9**	**5.4**	**4.6**
abuse	9.1	9.3	5	4.3
neglect	7.8	8.4	7.3	7.2
**KERF-40+ Multiplicity (number of subtypes)**	**4**	**4.3**	**2**	**2.4**
abuse	3.1	3.1	1.5	1.9
neglect	1	1.2	0.8	0.8

Supplementary Table S2: Additional Questionnaire Information on ACE History and Mental Health Status

**Childhood Trauma Questionnaire CTQ**		
	Mean	SD
CTQ_total	63.6	19.7
CTQ_EA (Emotional Abuse)	16.2	5.3
CTQ_PA (Physical Abuse)	9.7	4.8
CTQ_SA (Sexual Abuse)	10.4	6.3
CTQ_EN (Emotional Neglect)	17.4	5.4
CTQ_PN (Physical Neglect)	9.8	4.1
**Dissociative Experience Scale (DES. German Version – FDS)**		
FDS Total	13.8	12.2
DES Total	16.7	13.6
dissociative amnesia	6.7	8.5
absorption	23.2	16.3
derealization/depersonalization	14.2	18.7
conversion	9.1	12.5
**PTSD- Checklist for DSM-V (PCL-V)**		
intrusion	6.5	4.6
avoidance	3.6	2.5
neg. change in cognition and mood	10.2	6.8
hyperarousal	8	5.7
absolute symptom burden	28.3	17
**Brief Symptom Inventory**		
somatization	0.6	0.6
OCD	1.3	0.8
depression	1.2	0.9
anxiety	1.1	0.9
aggression	0.7	0.7
phobia	0.7	0.8
paranoia	0.9	0.7
psychotic	0.8	0.8
Global characteristics<br>- Global Severity Index (GSI)<br>- Positive Symptom Total (PST)<br>- Positive Symptom Distress Index (PSDI)	 0.9<br>26.9<br>1.7	 0.6<br>13.3<br>0.4

Supplementary Table S3: Psychiatric diagnoses of the participants based on SCID-5-CV

**diagnosis**	**lifetime (f/m)**	**current (f/m)**
PTSD	50 (45/5)	27 (25/2)
Affective Disorder<br>- of which Bipolar	66 (59/7)<br>none	27 (25/2)<br>none
Anxiety / OCD	45 (39/6)	35 (31/4)
Addictive Disorder	20 (16/4)	5* (5/0)
Eating Disorder	22 (22/0)	5 (5/0)
Somatoform Disorder	10 (8/2)	7 (5/2)
Diagnosis-Free	13 (10/3)	36 (29/7)
*last 12 months		

1.4.2 Random Forest

Supplementary Table S4: accuracy R^2 of all Random Forest models

model	**Amygdala**		**Hippocampus**		**ACC**	
	**left**	**right**	**left**	**right**	**left**	**right**
1	-0.093	-0.066	-0.087	-0.052	-0.002	**0.011**
2	-0.129	-0.075	-0.101	-0.107	-0.025	-0.048
3	-0.077	-0.008	-0.022	-0.0002	-0.073	-0.082
4	-0.067	-0.094	-0.039	-0.134	**0.011**	-0.025
5	-0.005	-0.003	-0.053	-0.047	-0.12	-0.078
6	-0.104	-0.071	-0.080	-0.083	-0.087	-0.03

1.4.2.1 Correlation Analyses of important variables

Supplementary Table S5: Correlation Analysis of the identified important Variables with the volume of the right ACC Pearson correlation coefficient r and significance value p.

		ACC right
KERF-40+ sum	r	-0.078
	p	0.458
KERF-40+ duration	r	0.037
	p	0.726

*Supplementary Table S6: Correlation Analysis of the identified important variables with the volume of the left ACC, Pearson correlation coefficient r and significance value p. * $p < 0.05$*

		ACC left
neglect Age 3	r	**0.246***
	p	**0.017**
neglect Age 4	r	**0.265***
	p	**0.01**
abuse Age 16	R	-0.087
	p	0.408

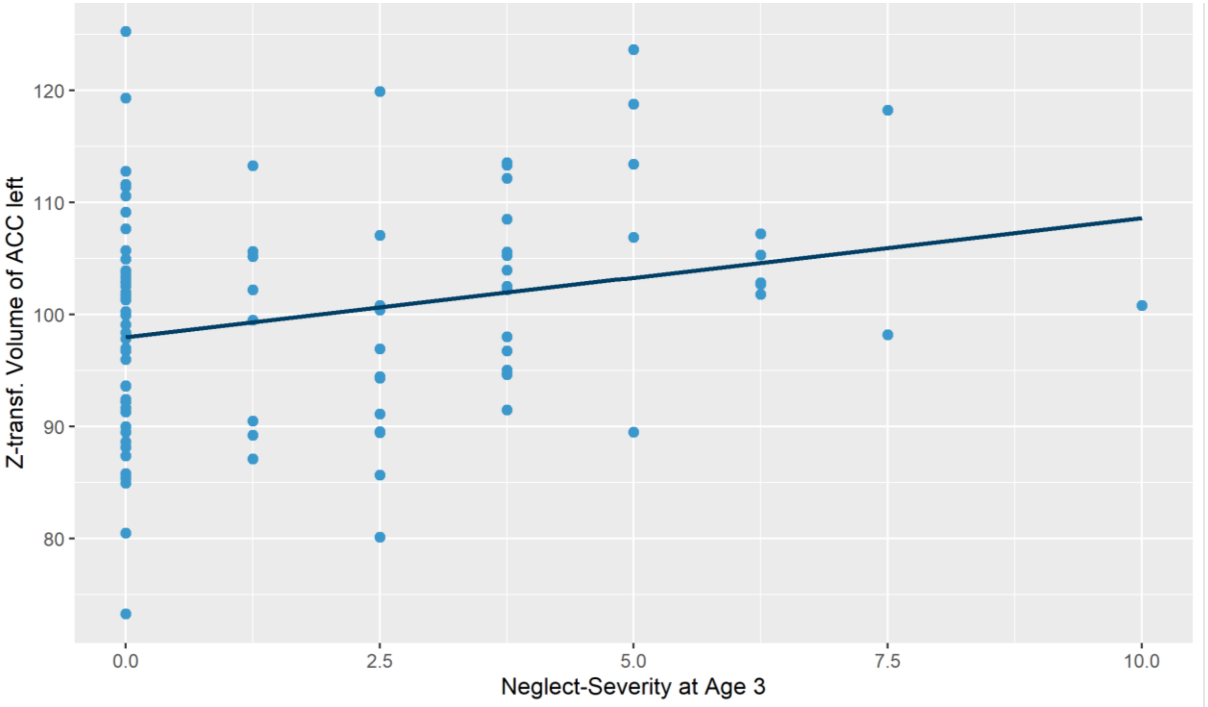

Supplementary Figure S1: Scatter plot for the relationship between neglect severity at age 3 and left ACC volume

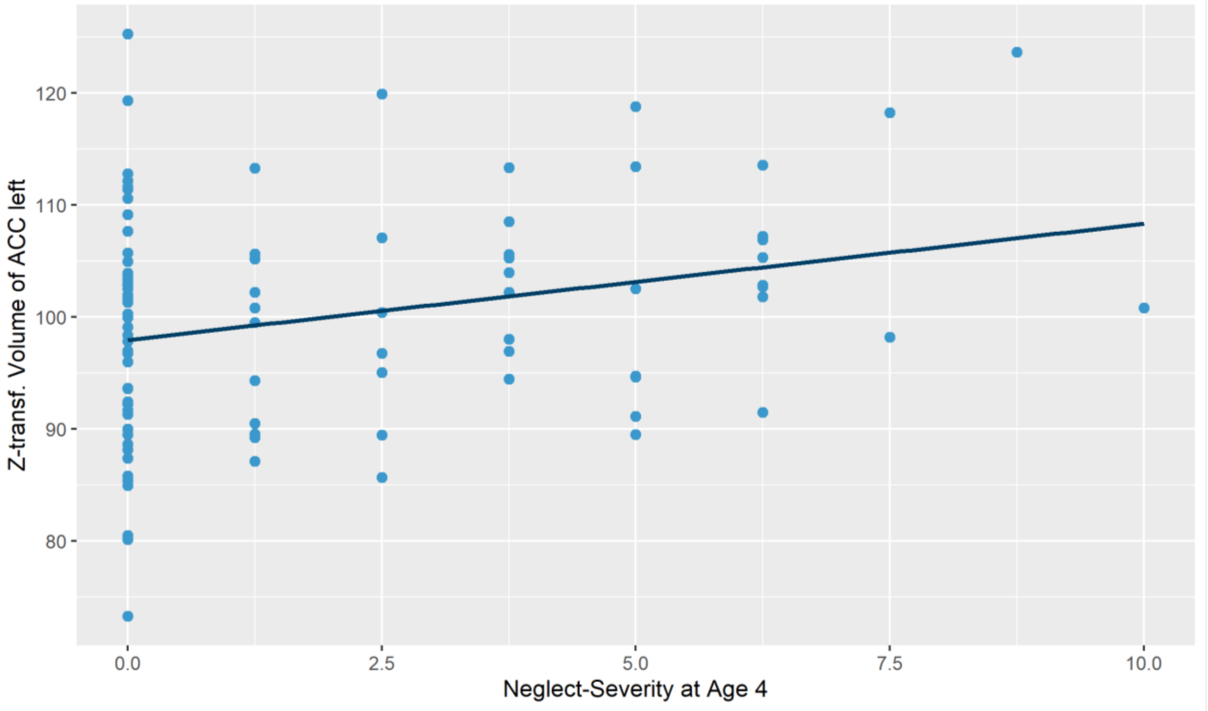

Supplementary Figure S2: Scatter plot for the relationship between neglect severity at age 4 and left ACC volume

1.5 References

1 Breiman, L. Random Forests. *Machine Learning* **45**, 5-32 (2001). https://doi.org:10.1023/A:1010933404324

2 Strobl, C., Boulesteix, A. L., Kneib, T., Augustin, T. & Zeileis, A. Conditional variable importance for random forests. *BMC Bioinformatics* **9**, 307 (2008). https://doi.org:10.1186/1471-2105-9-307

3 Altmann, A., Tolosi, L., Sander, O. & Lengauer, T. Permutation importance: a corrected feature importance measure. *Bioinformatics* **26**, 1340-1347 (2010). https://doi.org:10.1093/bioinformatics/btq134
